# Overexpression of a pine Dof transcription factor in hybrid poplars: A comparative study in trees growing under controlled and natural conditions

**DOI:** 10.1371/journal.pone.0174748

**Published:** 2017-04-04

**Authors:** Marina Rueda-López, María Belén Pascual, Mercedes Pallero, Luisa María Henao, Berta Lasa, Ivan Jauregui, Pedro M. Aparicio-Tejo, Francisco M. Cánovas, Concepción Ávila

**Affiliations:** 1 Departamento de Biología Molecular y Bioquímica, Facultad de Ciencias, Universidad de Málaga, Málaga, Spain; 2 Departamento de Ciencias del Medio Natural, Universidad Pública de Navarra, Pamplona, Spain; Youngstown State University, UNITED STATES

## Abstract

In this work, the role of the pine transcriptional regulator Dof 5 in carbon and nitrogen metabolism has been examined in poplar trees. The overexpression of the gene and potential effects on growth and biomass production were compared between trees growing in a growth chamber under controlled conditions and trees growing in a field trial during two growth seasons. Ten-week-old transgenic poplars exhibited higher growth than untransformed controls and exhibited enhanced capacity for inorganic nitrogen uptake in the form of nitrate. Furthermore, the transgenic trees accumulated significantly more carbohydrates such as glucose, fructose, sucrose and starch. Lignin content increased in the basal part of the stem likely due to the thicker stem of the transformed plants. The enhanced levels of lignin were correlated with higher expression of the *PAL1* and *GS1*.*3* genes, which encode key enzymes involved in the phenylalanine deamination required for lignin biosynthesis. However, the results in the field trial experiment diverged from those observed in the chamber system. The lines overexpressing *PpDof5* showed attenuated growth during the two growing seasons and no modification of carbon or nitrogen metabolism. These results were not associated with a decrease in the expression of the transgene, but they can be ascribed to the nitrogen available in the field soil compared to that available for growth under controlled conditions. This work highlights the paramount importance of testing transgenic lines in field trials.

## Introduction

The production of biomaterials and bioenergy is a process that has been used since ancient times by man in contrast with the main use of crop plants as a source of food. The growing demand for petroleum products, the limited reserves of fossil fuels and the global warming attributed to the use of this energy source indicate the urgent need to find alternative sources of renewable energy [1]. Biomass used for energy is mainly derived from the processing of agricultural and forest products, waste from holdings, the remains of forestry, and crop residues as well as from crops planted and exploited for the sole purpose of obtaining biomass [2]. These latter are called energy crops and include forest and agricultural crops. The fundamental advantage of such crops is the predictability of their layout and the spatial concentration of biomass, ensuring the supply of feedstocks for bioenergy. In the last 20 years, the biology of forest trees has been widely investigated, which is reflected in gradual biotechnological development aimed at cultivation on a large scale, as is the case with many plants of agronomic interest. However, the major genetic improvements that accompanied the agricultural practices have not yet occurred for trees. Although classical breeding approaches have much to offer in the field of tree improvement, applications of genomics and biotechnological methods can accelerate the process. In this sense, poplar is a fast growing tree that presents a number of logistical advantages and economic benefits over annual crops that can be used for similar purposes such as cereals. One of these benefits is its flexibility in harvest time, which reduces storage costs and losses associated with degradation of the stored crop biomass collected from annual crops. Other features that make poplar a good model for bioenergy crops are dehydration resistance, resistance to insects and other pests and the ability to produce large amounts of biomass in different soil types [3]. Furthermore, the poplar genome has been completely sequenced [4], and the availability of this information gives us a guide to the answers to many questions about growth and shape, disease resistance and quality of wood that would otherwise be much harder to address in trees studies.

The productivity of plants is hardly affected by nutrient availability. In field conditions, nutrient supply and nutrient limitation are closely linked [5]. One of the possible targets to increase poplar biomass production is to improve the absorption and metabolism of nitrogenous nutrients [6]. Nitrogen availability is, very often, one of the factors limiting plant growth, and the efficient use of nitrogenous nutrients is essential for the accumulation of biomass [7, 8, 9]. Previous results of our research group have shown that manipulation of the structural and regulatory genes involved in nitrogen metabolism may be a valid approach to increasing biomass production in hybrid poplars (*Populus tremula x P*. *alba*) and to producing wood with improved pulping attributes [7, 10, 11, 12, 13, 14].

The enzyme glutamine synthetase (GS) plays a key role in nitrogen assimilation in plants, a process closely coordinated with carbon metabolism because it requires the provision of carbon skeletons in the form of keto acids. The transcription factor Dof5 is able to regulate *GS* genes differentially in maritime pine. It acts as an activator of *GS1b* and as a repressor of *GS1a* [15]. Dof factors have been described as regulators of lignin production [16] and the carbon-nitrogen balance [13, 17]. The phenylpropane skeleton required for lignin biosynthesis is provided by deamination of phenylalanine catalyzed by the enzyme phenylalanine ammonia-lyase (PAL), and in this reaction, should be re-assimilated by GS to maintain the synthesis of lignin and other phenolic compounds [7]. These studies highlight the importance of Dof factors because of their involvement in the regulation of two main routes driving growth and development in plants: carbon and nitrogen metabolism.

Over the last decade, an increasing number of reports have been produced that propose genetic modifications for production improvements in plants, including poplar [18]. In addition, we can anticipate a perfect storm of studies targeting candidate genes in Populus spp in the coming years for the use of the CRISP/Cas9 technique [19, 20]. Currently, the new frontier of the genetic engineer is ensuring that the transgene incorporated in the plant is beneficial in field conditions, where the fluctuations in the environment make the plant performance realistic [21, 22]. Progressive testing of modified plants in natural conditions is mandatory for global crop improvement.

The manipulation of transcription factors to control multiple genes encoding products of the same or different pathways can be presented as an attractive and effective strategy to control the levels of metabolites of interest from both, qualitative and quantitative points of view [23]. In this work, the effects of the pine transcriptional regulator Dof 5 on carbon and nitrogen metabolism were examined in poplar trees. The overexpression of the gene and its potential effect on growth and biomass production were compared in young trees growing under controlled and natural conditions.

## Materials and methods

### Plant material, culture conditions and sampling

Hybrid poplar (*P*. *tremula x P*. *alba*), clone INRA 7171-B, Institut National de la Recherche Agronomique, INRA), was transformed via *Agrobacterium tumefaciens* strain C58C1 containing the binary plasmid vector pBi121 with the cDNA of *PpDof5* (accession number AM884254). The transformation method used was as previously described [24] and transformed explants were selected with kanamycin (30 mg L^-1^) and maintained in a controlled chamber at 24°C, and 16h light photoperiod. Hybrid non-transformed poplar plants were used as control.

Before *ex vitro* culture, shoots were rooted on one-half strength MS medium supplemented with 0.5 mg L^-1^ indole-3-acetic acid as previously described [24]. The transgenic and control plantlets (average 8–10 cm) were transferred to pots for acclimatization and growth in a controlled growth chamber (KoxKa, Spain). First, plants were transferred into plastic pots contained vermiculite and covered with plastic bags during 2 weeks. Later, plants were transferred to pots containing a potting mix and vermiculite in a 3:1 proportion and grown for 10 more weeks. After this time, each plant was divided into different sections: the apical leaves, from 1st to 6th leaf starting from the shoot apical meristem; the basal leaves, from 13th to 18th leaf from the shoot apical meristem; the stem; and the principal root. All the samples were frozen in liquid nitrogen, reduced to powder with a mixer mill MM400 (Retsh, Germany) and stored at -80°C.

For the field trial, transformed poplar lines and controls micropropagated *in vitro*, were acclimatized in a growth chamber as described before. They were further acclimatized in a greenhouse for 16 weeks from July to October before they were transferred to the field by the end of October. The experimental design in the field was conducted by distributing lots of 5 plants of lines and controls in three replicates. The lots were planted at random. The replicates were distributed in three rows with 40 plants per row. The distance between planting rows was 350 cm. The distance between trees was 50 cm. The test was surrounded by two rows of unmodified poplar trees to avoid edge effect. The field trial test was carried out during two years in a region of Navarra (Spain). Samples were harvested in May of the two following years. In May of 2012, upper leaves (leaves in the first meter of height of the tree, from the apical meristem), basal leaves (leaves at different heights between the first and second meter from the apical meristem) and side branches were harvested. In May 2013, leaves (between 1.5–2.0 m from apical meristem) and side branches were harvested. Samples were frozen in liquid nitrogen and reduced to powder and stored at -80°C.

### RNA extraction and RT-qPCR analysis

RNA extraction and real-time quantitative PCR were performed and analyzed as described previously [12]. To determine the level of expression of *PpDof5* in transgenic plants, the primers used were Dof5-F/Dof5-R (S1 Table).

A set of poplar genes was also analyzed by RT-qPCR: Glutamine synthetase gene, *PtGS1*.*3*, Phenylalanine ammonia-lyase gene *(PAL)*, *PtDof4* and *PtDOf19* were amplified and measured as described previously [25]. The expression was analyzed using the following primers: PtPAL-F/PtPAL-R. The ubiquitin gene (accession number BU879229) and Actin2 (BU879695) were used as reference genes [26]. All primers are included in S1 Table.

### Determination of transgene copy number

The copy number of the transgene in the genome of *Populus* was determined by Southern blot and by PCR [27]. As an endogenous single copy gene, we selected FAD-dependent oxidoreductase (Potri.018G112600) [28]. Genomic DNA was isolated with the CTAB method [29] and quantified with a NanoDrop ND-1000A UV-Vis spectrophotometer (Thermo Fisher Scientific, USA). One hundred nanograms of genomic DNA of each plant sample were used for amplification using Biotools DNA polymerase (Biotools, B&M Labs, Madrid Spain) and primers pcrFAD-F/pcrFAD-R and pcrDof5-F/pcrDof5-R (S1 Table). After 25 cycles, the PCR products were tested on an agarose gel and visualized with ethidium bromide. The gel image was captured using a ChemiDoc XRS+ System (BioRad) and the intensity of the band analyzed with the ImageJ software [30].

To determine whether the primers for PCR had the same amplification efficiency, both the endogenous gene and the PpDof5 ORF were amplified with iProof HF Master Mix (BioRad, USA) using the primers PtFAD-F/PtFAD-R and PpDof5-F/PpDof5-R, respectively. The PCR products were cloned in pJET1.2/blunt using a CloneJET PCR cloning Kit (Thermo scientific, USA). RT-qPCR was carried out in a thermal cycler CFX384 (BioRad, CA, USA) using SsoFast™ EvaGreen® Supermix (BioRad, CA, USA), 20 ng of plasmid DNA and primers pcrFAD-F/pcrFAD-R or pcrDof5-F/pcrDof5-R under the following conditions: 3 min at 95°C (1 cycle) and, 1 s at 95°C and 5 s at 60°C (40 cycles).

The Southern blot analysis was carried out basically as described before [31]. Ten micrograms of poplar genomic DNA from each transgenic line and control plants were digested with *Bam H1*, electrophoresed and transferred. The blot was hybridized against the pine Dof5 cDNA labeled probe.

### Carbohydrate content

Soluble carbohydrates were extracted from 100 mg of tissue using 500 μL of 80% ethanol at 80 ^0^C for 30 min, followed by further washing with 250 μL of 50% ethanol at 80 ^0^C for 20 min and two additional washes with 250 μL of water. Combined supernatants were lyophilized, and the resulting powder was suspended in water and used to determine enzymatically glucose, fructose and sucrose levels as described previously [32]. The pellet obtained, after washing with ethanol, was dried to 60 ^0^C and used to determined starch level [33].

### Determination of cellulose and lignin

Cellulose content was determined by the anthrone colorimetric method [34] with modifications for poplar samples [14]. Lignin levels were determined by the thioacidolysis method [35] and adapted as previously described [36].

### Histochemical staining of lignin

Stem pieces (5 mm-length) corresponding to internode 12–14 from the apical meristem were fixed with 1.25% (v/v) glutaraldehyde in methanol by the freeze substitution method [37]. Tissue pieces were embedded by immersing them sequentially into 100% methanol, 100% ethanol, 3:1 (v/v) ethanol: Histolemon (Carlo Erba, France), 1:1 (v/v) ethanol: Histolemon, 1:3 (v/v) ethanol:Histolemon, Histolemon and Histolemon: Paraplast X-tra (Leica, Germany). All steps were performed at room temperature and under continuous agitation. Finally, the Histolemon: Paraplast X-tra mix was replaced with pure liquid Paraplast X-tra at 62°C. Sections of 10 μm were cut using an RM2125 RTS manual microtome (Leica) and mounted on poly-L-lysine-coated glass slides (Menzel-Gläser, Germany). Sections were deparaffinized and hydrated sequentially through an ethanol series. Staining was performed with 1% phloroglucinol in 2:1 ethanol absolute:HCl and images were captured with an AZ100 Multizoom Microscope (Nikon, UK)

### Determination of chlorophyll

The extraction of total chlorophyll was performed from 100 mg of frozen samples in liquid nitrogen and 80% (v/v) acetone, and the content was assessed as previously described [38].

### ^15^N uptake in hydroponic culture

In vitro rooted transgenic and control plantlets were acclimatized in transparent plastic pots (500mL volume) containing Hoagland solution [39] for 2 weeks. After this period, the plants (average height 10–15 cm) were irrigated with a modified Long-Ashton medium [40]. The plants were grown first with complete medium for 2 weeks and later with medium containing a single source of inorganic nitrogen (either 1 mM KNO_3_ or 1 mM NH_4_Cl) for 5 weeks more [41]. Throughout the experiment, roots were in darkness and conditions were the same as described previously.

To determine the uptake of nitrogen, plants were incubated for 2 h in the same solution but containing ^15^N-label (either as 1 mM K^15^NO_3_ or 1 mM ^15^NH_4_Cl). Later, roots were washed with 0.5 mM CaCl_2_ to remove any adsorbed ^15^N. Roots were oven-dried at 70°C for 2 days and ground into powder for the analysis of ^15^N content. δ^15^N (‰) values were determined using a Flash EA 1112 elemental analyzer coupled to a Delta V Advantage isotope ratio mass spectrometer (Thermo Scientific, USA). Nitrogen influx was determined as described previously [42].

### Determination of N content in soil samples

Nitrogen content was determined in dry samples of soil previously ground to powder. One-milligram samples were stored in tin capsules. Nitrogen content was determined using a CNS 2500 elemental analyzer (CE Instruments, Italy).

### Statistics

The lengths of plants and roots were measured and ^15^N uptake analyzed in five plants by line. The mean +/- standard error of the mean is shown. For the RT-qPCR and the, determination of levels of carbohydrates, lignin and enzymatic activity, three independent biological replicas were measured in triplicate, and the values shown are the mean +/- standard error. In all cases, asterisks indicate that the difference between the control and transgenic plants was significant by the t-test (ρ < 0.05).

## Results

### Transformation and characterization of poplar transgenic lines growing under controlled and natural conditions

**Fig 1:**
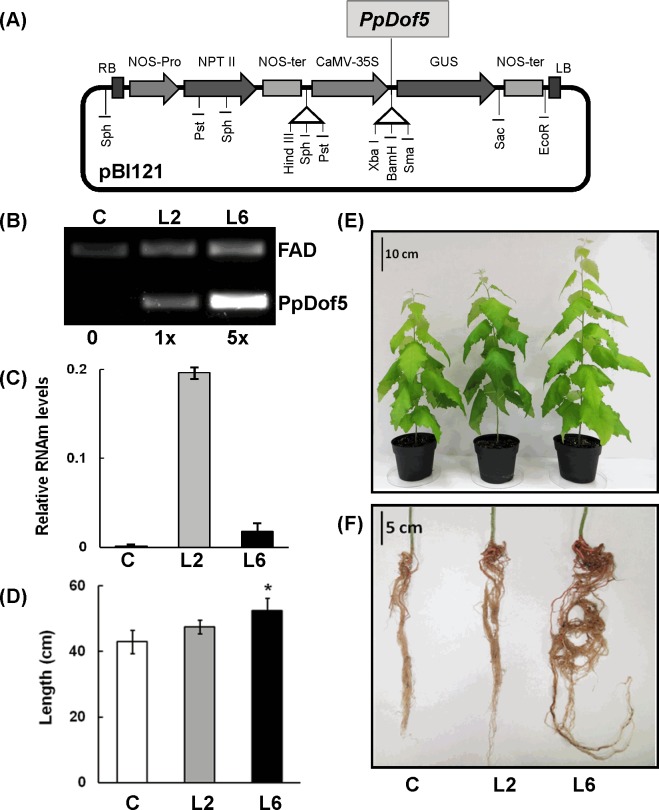
Analysis of the transgenic poplar lines. (A) Diagram of the construct used to transform hybrid poplar clone INRA 7171-B *(P*. *tremula x P*. *alba)*: the pBI121 vector containing the *PpDof5* sequence under the control of a 35S gene promoter (CaMV35S) was used for the transformation. (B) Determination of copy number in the L2 and L6 transgenic lines using quantitative PCR. The poplar single copy gene FAD-dependent oxidoreductase (Potri.018G112600) was used as the standard. The ratio of the amplicons derived from the integrated *PpDof5* and the internal control is indicated at the bottom of each line. (C) Expression analysis of *PpDof5* by real-time qPCR in controls (white bar) and two transgenic lines: L2 (gray bar) and L6 (black bar). The expression data were normalized using ubiquitin as the reference gene. (D) Representative picture of 10- week-old untransformed control and L2 and L6 transgenics. (E) Root volume of control and transgenic poplar plants. (F) Length of the stem in cm of the plants (five plants were used for each determination).

A fragment of 1700 bp containing the cDNA of *PpDof5* (AM884254) was inserted into the binary vector pBI121 under the CaMV 35S constitutive promoter (Fig 1A) and used to transform hybrid poplar *(P*. *tremula x P*. *alba)* leaf discs. Transgenic lines were produced and maintained *in vitro* as previously described 43] until they were transferred to plastic pots and grown in a growth chamber for 10 weeks. Twelve independent lines were analyzed for the presence of the transgene. The copy number was determined by Southern blot analysis (S1 Fig). Two transgenic lines were selected for further characterization on the basis of the number of gene copies present in their genomes, a single copy for L2 and multiple copies for L6 (Fig 1B). These two lines exhibited different expression levels of the transgene as determined by qPCR analysis of three individuals from each transgenic line (Fig 1C). The *PpDof5* expression levels observed in L6 were approximately 10 times lower than those detected in L2.

**Fig 2:**
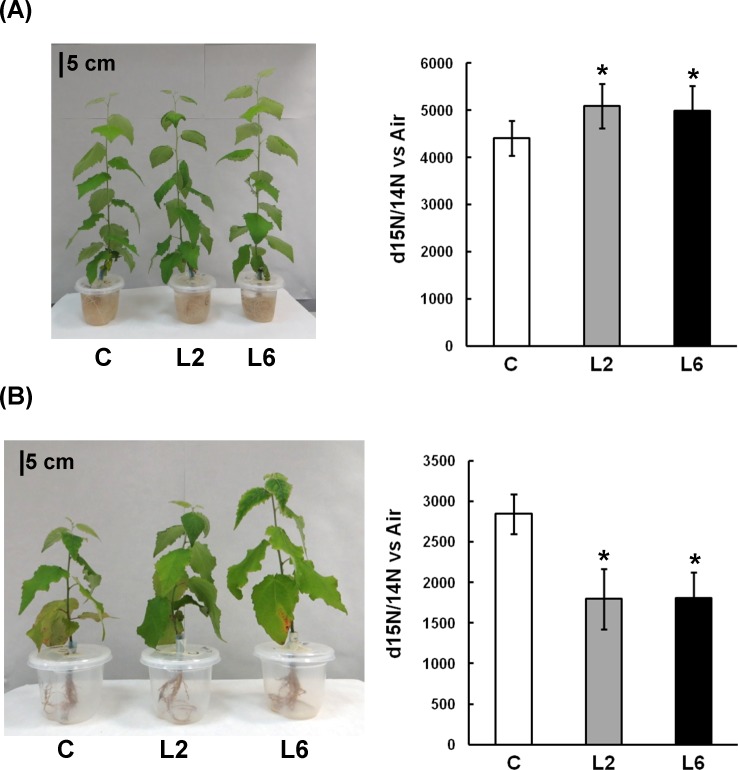
^15^N uptake in hydroponic culture. (A) Plants acclimatized in transparent plastic pots as described in Materials and Methods and grown in KNO_3_ as the sole N source were incubated for 2 h in the same solution but containing ^15^N-label as 1 mM K^15^NO_3_. δ^15^N (‰) values were determined in roots using an elemental analyzer coupled to a Delta V Advantage isotope ratio mass spectrometer. Values are in triplicate, and asterisks indicate that the difference between the control and transgenic poplars is significant by the *t*-test (P<0.05).(B) The same as in (A), except that plants were grown under NH_4_Cl as the sole N source.

To examine the impact of *PpDof5* overexpression in transgenic hybrid poplar, growth performance was examined in transgenic and untransformed controls. The length of five plants from each line and the control grown under controlled conditions in a growth chamber was measured. Overall, the transgenic plants showed higher vegetative growth in the aerial part (Fig 1D) as well as increased root volume (Fig 1E). Quantitatively, slight differences were observed for L2 plants from the average of untransformed controls, while transgenic line L6 exhibited a general greater growth, resulting in significant increase in height (Fig 1F). The observed increase in root volume suggested potential differences between transgenic and control untransformed plants in nutrient uptake. To further investigate this possibility, 10-week-old poplar plants were adapted to a hydroponic system and used to test ^15^N content and their preferred nitrogen source when they were cultured in medium containing either KNO_3_ or NH_4_Cl as the sole nitrogen source. Fig 2 shows that control plants preferred nitrate over ammonium as a nitrogen source. Nitrate uptake in the transgenic lines (L2 and L6) significantly increased compared to controls (Fig 2A). When ammonium was used as the sole N source, plant growth was affected and the ^15^N uptake in the lines was significantly decreased compared with controls (Fig 2B).

**Fig 3:**
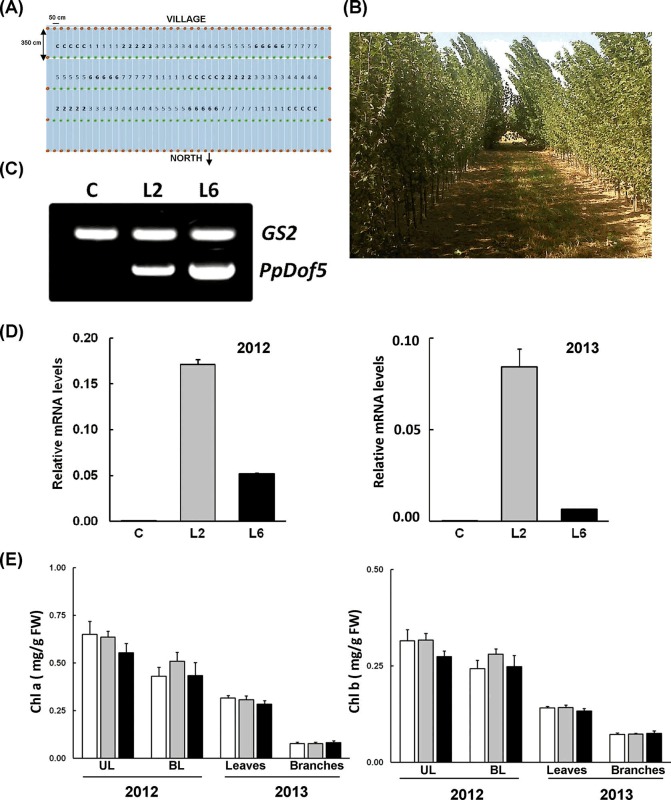
Field trial of transgenic poplar overexpressing *PpDof5*. (A) Diagram of the ground for the field trial experiment. (B) General overview of ground-field trees in the field trial. (C) Confirmation of the presence of the transgene in field-growing trees. PCR analysis was performed using primers specific for *PpDof5* on genomic DNA from poplar. As a control, the internal poplar gene *PtGS2* was amplified. Two kinds of samples from years 2012 and 2013, the two growing periods considered, were analyzed. (D) The mRNA level of *PpDof5* was determined by qPCR in leaves of field-growing trees in 2012 and 2013. Determinations were made in triplicate. (E) Content of chlorophyll a and b in controls and transgenic poplar was determined in the upper (UL) and basal (BL) leaves of trees during the growing period of 2012 and in leaves and branches during the growing period of 2013. White bars correspond to control samples, gray bars are L2 and black bars are L6 samples.

Considering that an increase in biomass is one of the main targets of genetic engineering in forestry and given the promising performance of the *PpDof5* transgenic lines during the initial period of growth, we decided to begin a field experiment. A field trial test was established in the region of Navarra (Spain) in 2011 (Fig 3A). The trial was approved by the Spanish Comision Nacional de Bioseguridad, Ministerio de Medio Ambiente (B/ES/11/26). One hundred and twenty rooted plants with an average height of 1m corresponding to control and seven independent transgenic lines were planted in the plot and grown for two years (Fig 3B). The field trial design is fully described in the Materials and Methods section. Three individuals each of untransformed controls, L2 and L6 were used for biochemical determinations over two growing seasons, during 2012 and 2013.

The presence of the transgene in the genome of the poplar trees was confirmed by isolation of genomic DNA and PCR amplification (Fig 3C). The expression of *PpDof5* was stable during the two years of the experiment, and the relative levels of transcripts for lines L2 and L6 were quite similar to those observed during early growth under controlled conditions (Fig 1C). The transgenic plants of Line 6 exhibited lower transcript abundance than Line 2 plants in 2012 and 2013 (Fig 3D). No growth differences were observed among the trees after one year in the field or after two years. Chlorophyll a and b contents were also similar between control and transgenic plants and remained without significant changes for the two growing seasons (Fig 3E).

### Effect of *PpDof5* overexpression on the soluble sugar content of hybrid poplar

**Fig 4:**
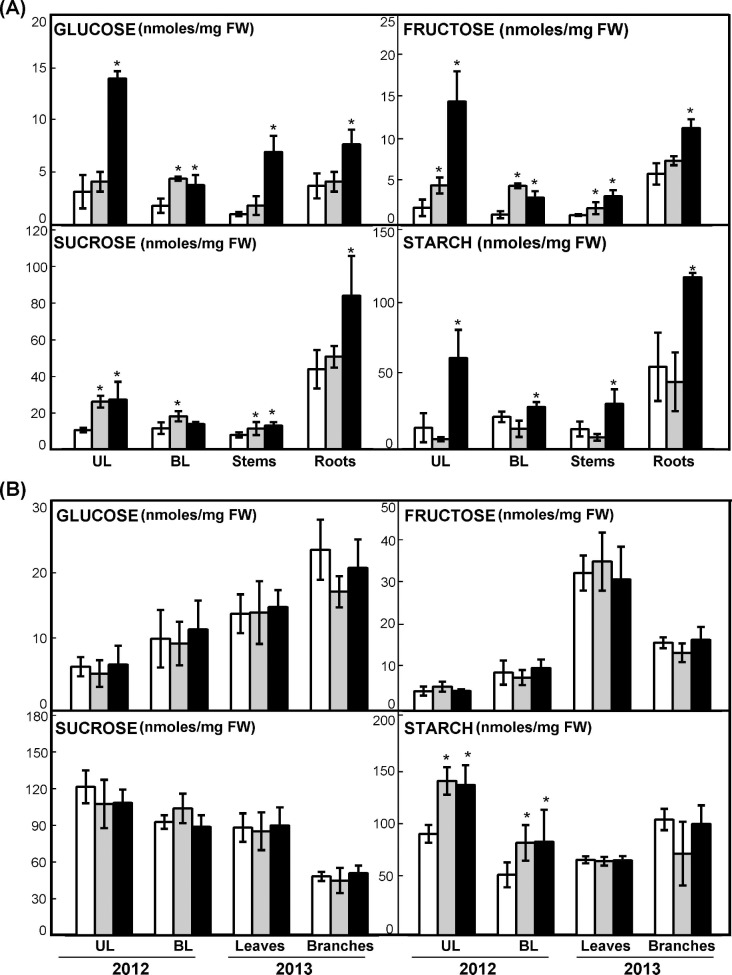
Carbohydrate content in *PpDof5* transgenic poplar. (A) Analysis of carbohydrate content in 10-week-old control and transgenic poplar lines grown in the growth-chamber. Values are expressed as nmol mg^-1^ of fresh weight (FW). The determination is the mean of three independent biological samples measured in triplicate in upper leaves (UL), basal leaves (BL), stems and roots. Asterisks indicate that the difference between the control and transgenic poplars is significant by the *t*-test (P<0.05). (B) Analysis of carbohydrate content in control and transgenic poplar lines grown in the field during the two-growing seasons of the experiment. Values are expressed as nmol mg^-1^ FW and are the means of three independent biological samples measured in triplicate. The samples were upper leaves (UL) and basal leaves (BL) for 2012 and leaves and branches for 2013. Asterisks indicate that the difference between the control and transgenic poplars is significant by the *t*-test (P<0.05) for starch content in samples of 2012.

Considering that the overexpression of *PpDof5* increases soluble sugars content in *Arabidopsis* [13], the levels of soluble sugars were determined in the transgenic trees. The glucose, fructose, sucrose and starch contents were measured in the upper and basal leaves (source tissues), the stem and the principal root (sink tissue) of 10-week-old plants grown under controlled conditions in a growth chamber. There were significant differences in soluble sugars content between lines L2 and L6 and with regard to control plants. Overall, the glucose and fructose contents were significantly higher in all tissues of L6 plants compared to control plants (Fig 4A, glucose and fructose). Glucose content was higher only in the basal leaves of L2 plants (Fig 4A, glucose). We should note that the increase detected in the glucose content was significantly higher in all L6 tissues compared to L2, except for the basal leaves where the glucose content is parallel and higher than in control plants.

Sucrose content was significantly higher in the upper leaves and stems of both lines. Further, sucrose content was also higher in the roots of the L6 line, where the observed values were approximately 4-fold those detected in leaves (Fig 4A, sucrose). Again, these differences were more significant for the different sections of L6 trees.

Starch is a major storage carbohydrate in plants, and it has been identified as a major integrator in the regulation of plant growth to cope with continual changes in carbon availability [44]. Increased amounts of starch were observed in all tissues examined in the L6 line, particularly in the roots. In contrast, no major differences were found in the L2 line (Fig 4A, starch). This higher starch content in L6 parallels the greater vegetative growth described for L6 plants grown under controlled conditions (Fig 1D–1G).

The glucose, fructose, sucrose and starch contents were measured in samples harvested from transgenic trees growing in the field trial under natural conditions. No significant differences were observed in the contents of glucose, fructose or sucrose in the upper and basal leaves and branches of *PpDof* transgenics compared to untransformed controls (Fig 4B, glucose, fructose, sucrose). Although increased content of starch was observed in the upper and basal leaves from the L2 and L6 lines during the first growth season (Fig 4B, starch), such differences were not found in the leaves and branches from the second year of the study.

### Cellulose and lignin content in poplar trees overexpressing *PpDof5*

**Fig 5:**
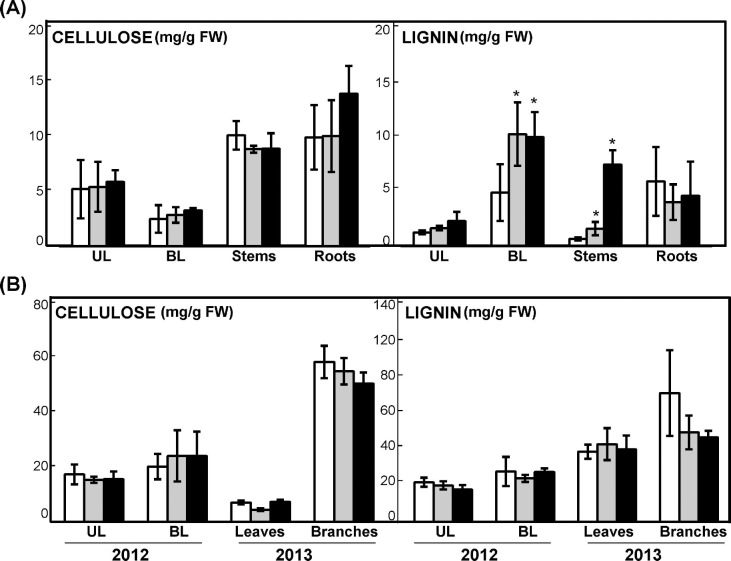
Cellulose and lignin content in *PpDof5* transgenic poplar. (A) Determination of cellulose and lignin content in 10-week-old poplar plants grown in a growth-chamber. Values are expressed as mg/g FW. The determination was performed in triplicate in three independent biological samples. Asterisks indicate significant differences between controls and lines by the *t*-test (P<0.05). (B) Determination of cellulose and lignin content in poplar trees grown in the field. Values are expressed as mg/g FW. The determination is the mean of three independent biological samples measured in triplicate.

To further characterize the biological performance of the transgenic plants, the contents of cellulose and lignin were compared during growth under controlled and natural conditions. As expected, the amount of cellulose was higher in the stems and roots of chamber-grown trees compared to leaves (Fig 5A). Similarly, branches of trees growing in the field contained more cellulose than leaves in the two lines L2 and L6 (Fig 5B). However, cellulose content was not significantly affected by the presence of the transgene in any of the samples analyzed. In contrast, chamber-grown trees of the L2 and L6 lines accumulated greater lignin content in basal leaves and stems compared to untransformed controls (Fig 5A). Interestingly, this enhanced accumulation was not observed in samples from trees grown under natural conditions during the two periods of growth examined (Fig 5B).

The enhanced accumulation of lignin in transgenic trees was further examined by histochemical analysis of stems of control and transgenic lines (S2 Fig). Phloroglucinol staining of transversal cross-sections revealed lignin deposition in the vascular tissue. The lignin distribution pattern displayed no differences among control and transgenic lines, and sections of the basal part of the stem showed identical pattern content and distribution among samples (S2 Fig). However, it is worth mentioning that the vascular cylinder in the over-expressing lines was routinely wider in the transgenic plants (L2: 903 μm ± 8.6; L6: 973μm ± 6.6) than in control plants (816 μm ± 37.2)

### Accumulation of lignin in the stems of the *PpDof* transgenics is correlated with higher expression of the *PAL1* and *GS1*.*3* genes

**Fig 6:**
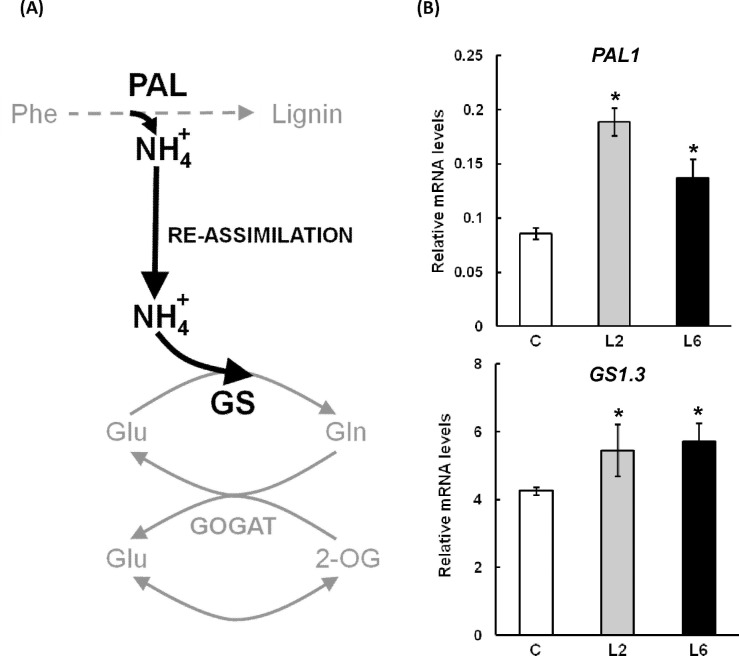
Expression analysis of *PAL1* and *GS1*.*3*. (A) Diagram of the location in the pathway of the two enzymes involved in the synthesis of phenylalanine for lignin synthesis. The ammonium flux involving the two enzymatic reactions is highlighted in black. (B) Expression analysis by qPCR of *PAL1* and *GS1*.*3* genes in the basal section of the stem in control and transgenic poplar trees. Measurements were performed in triplicate in three independent biological replicas. Asterisks indicate that the difference between the control and transgenic poplars is significant by the *t*-test (P<0.05).

In lignin biosynthesis PAL enzyme provides the phenylpropane skeleton and releases ammonium that is reassimilated by GS (Fig 6A). In poplar, the *GS* gene family is organized in four groups of duplicated genes, three of them encoding cytosolic GS isoforms and one encoding a chloroplastic isoform [25]. A previous study indicates that *GS1*.*3* mRNA is highly abundant in the vascular bundles of the stems suggesting that the encoded GS isoform is involved in the reassimilation of the ammonium released by PAL [45]. The *PAL* gene used for this study was selected using the http://popgenie.org/exnet tool [46]. Potri.016G091100 and its duplicate are the unique members of the *PAL* family expressed in wood and are therefore highly expressed in stems. The gene was named *PAL1* in this work. Fig 6B shows that the transcript levels of the *GS1*.*3* and *PAL1* genes were significantly higher in the stem sections of the transgenic lines.

The present study indicates that the performance of transgenic trees overexpressing *PpDof5* growing under natural conditions did not match the performance of the plants in the growing chamber, where more growth, increased carbohydrate content and biomass accumulation were observed. It has been described that plants phenotypes are shaped by the interaction between genotype and the availability of essential nutrients [47, 48]. Furthermore, N fertilization has a significant effect on growth, biomass and wood chemistry in poplar [49]. Taking this into account, it was of paramount importance to determine N availability for trees in both conditions. The N content in soil samples was determined using an elemental macro-analyzer. Soil samples from the field experiment had N total content twelve times lower than that of soil samples from the growth chamber experiment (Table 1).

**Table 1:** Total N determination of soil samples.

Sample	N in mg/100mg of soil
**Field soil**	0.13 +/-0.01
**Potting mix**	1.57+/-0.08

Nitrogen content was determined in dry samples of soil previously ground to powder using a CNS 2500 elemental analyzer. The determination was performed in triplicate.

## Discussion

Growth, biomass production, trunk shape, and wood quality are important features of interest for improving trees. Current research has been focused on several aspects of metabolism that will contribute to improving these characteristics and the study of key genes and gene networks determining the required phenotypes [50, 51, 52]. In this context, the manipulation of a single transcription factor to influence growth and biomass production seems an attractive method to achieve this goal.

Here, we report the generation of transgenic poplar trees overexpressing a pine Dof transcription factor and the plants performance under controlled and natural conditions, characterizing their molecular and physiological behavior.

The aim of this work was to manipulate simultaneously nitrogen and carbon metabolism in poplar trees using a single gene. Two lines (L2 and L6) with different copy numbers (Fig 1B) and different levels of transgene expression were selected to compare the performance of transgenic plants growing under controlled and natural conditions. Overall, the two lines had a analogous behavior, although the observed differences with respect to the controls were always more significant in trees of the L6 line. Multiple insertions of the transgene in the poplar genome led to silencing of *PpDof5* expression in comparison with a single transgene insertion. Interestingly, the basal levels of the transgene product had a more noticeable general effect on plant growth. These results suggest that high-level expression of *PpDof5* could affect the activity of endogenous poplar Dof genes, tending to minimize the transgene effect A search in the poplar genome allowed the identification of 41 *Dof* genes (S3 Fig). Three genes were identified as putative ortologues of *PpDof5* including: a pair of duplicated genes, *PtDof4* and *PtDof14*, and a single gene, *PtDof19*. The level of expression of *PtDof4* and *PtDof19* were measured in stems and leaves of plants grown both in the growth-chamber and in the field (S4 Fig). The presence of the transgene affected differentially the expression of *PtDof4* and *PtDof19* in the transgenic lines. These results clearly indicate an effect of the transgene insertion in the expression of the endogenous poplar genes and the differential effect can be related with the different performance of the two lines.

Ten-week-old *PpDof5* transgenic poplars increased in height, leaf number and root biomass. However, the increases in growth were much lower than those observed in hybrid poplars overexpressing a cytosolic glutamine synthetase (GS1) [43]. Moreover, the improved growth was associated with enhanced nitrate uptake in the transgenic lines. Poplars have a family of high-affinity and low-affinity nitrate transporters encoded by a large gene family [53] that account for nitrate acquisition by the trees. The overexpression of *Dof 5* could be responsible for the activation of nitrate transporters. In fact, it has been previously shown, following a functional genomics approach, that the overexpression of Dof transcription factors in cell cultures of *Arabidopsis* affects either positively or negatively the expression of several nitrate transporters [54]. The significantly higher levels of nitrate uptake observed in the present work suggest that activation of some of these transporters led to increased nitrogen uptake, resulting in an advantage for vegetative growth in these plants.

In contrast to the higher capacity for nitrate acquisition, the young transgenic trees exhibited decreased ammonium uptake and arrested growth. In fact, the use of ammonium as the sole nitrogen source arrested the growth of both transgenics and controls compared to those supplied with nitrate as the sole nitrogen source. These findings are consistent with the preference for nitrate over ammonium that many poplar species exhibit (Castro-Rodríguez, unpublished). Ammonium uptake is mediated by ammonium transporters (AMT) that are strategically located primarily in poplar root cells. A family of 16 ammonium transporters (AMTs) has been identified in the *P*. *trichocarpa* genome sequence by *in silico* analysis [55, 56]. The decrease in ammonium uptake in the transgenics could be explained by the regulation of some of these AMT genes in the plants overexpressing *Dof5* as previously described [54,57].

Carbohydrate content was higher in 10-week-old transgenic trees as previously observed in *Arabidopsis* over-expressing *PpDof5* [13]. Growth of plants depends on photosynthetic activity, and a larger number of leaves implies greater accumulation of carbohydrates that can be used for growing. Sucrose is the major stable product of photosynthesis transported from leaves to the growing parts of the plant [58]. The increased availability of sucrose in developing parts of the tree can therefore contribute to increased growth. Starch accumulation was also significantly higher in the L6 line, at least at the time of day when the samples were collected (4 h after starting light). Considering that carbon availability is variable during the day, the higher accumulation of starch during the day-time provides a greater amount of C to support tree growth either during the day or the night, although it has been shown that it is not starch itself but starch metabolism or signals derived from starch that can act as integrators of plant metabolism and growth [44].

The measurement of cellulose content did not reveal significant changes in the trees, only the faster growth contributes to the greater thickness of the stem cambium, which is apparently responsible for the higher content of lignin. This higher lignin content is in good agreement with the expression of the *PAL1* and *GS1*.*3* genes in this region of the stem. These findings suggest that the higher level of expression of these two genes could account for the increased levels of phenylalanine in the *PpDof5* transgenics that are required for vascular development. Similar results have been described in other plants overexpressing *GS* genes [11,13,59].

Moreover, the increased levels of *GS1*.*3* transcripts in transgenic poplars are in agreement with the previously observed effect of *PpDof5* as a transcriptional activator of expression of the *GS1b* gene, an orthologous GS gene in pine [15]. The expression of the *GS1b* gene in pine is located within the vascular system in pine seedlings [59] and in the procambial cells of developing pine embryos prior to the differentiation of the vascular elements [60]. The function of *PtGS1*.*3* associated with nitrogen recycling during lignification in poplar [25] could be similar to that of *GS1b* in pine.

A field trial of the transgenic trees was performed during two growing seasons

It represents a suitable way to check the behavior of trees overexpressing *PpDof5* in the field, as growing conditions throughout a year do not always mimic the conditions in a growth chamber. A number of variables that do not affect the plants in the growth-chamber such as wind, rain, nutrient availability or biotic interactions with other organisms in the field can greatly modify trees growth. However, even though an ample number of structural and regulatory genes with potential impact on biomass production have been analyzed in transgenic trees, field-testing is lacking for most of those genes [51]. The data presented here derived from the field study are largely at odds with previous results obtained in growing chambers. Tree growth was not higher in the transgenics, and the contents of carbon compounds, such as chlorophyll, carbohydrates, cellulose and lignin, did not change when controls and transgenics were compared. In contrast, hybrid poplars over-expressing the structural *GS1a* gene from pine displayed considerable improvements in biomass production under both controlled and natural conditions [10]. Nevertheless, it is worth mentioning that there are several examples in which the phenotype observed in transgenic trees in growth chambers differed from that observed in field trials [61,62]. In our field-test it was found a meaningful difference between the study under controlled conditions and the field trial was the availability of nutrients in the soil. The analysis of nitrogen in soil indicated that the field study was carried out with a lower nitrogen content (approximately 12-fold) compared to the study carried out under controlled conditions. Therefore, the results indicate that the effect of Dof5 transgene expression seems to be relevant to growth and development only when N availability in the soil is sufficient. These data again highlight the close relationship between the metabolism of carbon and nitrogen and the role of Dof5 in the regulation of nitrogen/carbon balance. Furthermore, this study reinforces the paramount importance of field evaluation of transgenic plants, which is essential to determining their true value, especially in the case of trees that have to cope with long growing periods.

## Supporting information

S1 Table Primers used in this study. (DOCX)

S1 Fig Southern blot analysis of independent transgenic *PpDof5* lines. 10 μg of genomic DNA from control plant (C) and each line (L1-L10, L12 and L13) were digested with *BamHI* and separated on an agarose gel. Blots were hybridized with ^32^-P labelled PpDof5 cDNA.
(TIF)

S2 Fig Histochemical analysis of lignin in basal sections of the stem. Cross-sections (10 μm thick) of control and transgenic lines were processed as described in Materials and Methods. The sections of stem were stained with phloroglucinol-HCl solution for detecting lignin.
(TIF)

S3 Fig Phylogenetic analysis. Phylogenetic tree of the deduced protein sequences of genes encoding Dof transcription factors from: *Arabidopsis thaliana* (36 sequences), *Oryza sativa* (31 sequences), *Populus trichocarpa* (41 sequences) and *Pinus pinaster* (10 sequences). As out of group were used two Dof sequences from *Physcomitrella patens*. The proteins are classified in subfamilies A-G. PpDof5 is classified in subfamily B.
(TIF)

S4 Fig Expression analysis of *PtrDof4* and *PtrDof19*. (A) Transcript levels were determined in stem (S) and leaves (L) of 10-weeks-old control hybrid poplar and L2 and L6 transgenics plants grown in a growth-chamber by qPCR using specific primers (S1 Table). (B) Transcript levels in side branches (B) and leaves (L) of poplar trees grown in the field. The expression data were normalized using *Ubiquitin* and *actin* as reference genes. Data represent the mean ± standard error of three technical replicates. Asterisks indicate that the difference between the control and transgenic plants was significant by the t-test (P< 0.05).
(TIF)
