# Supplementary material for: Overexpression of a pine Dof transcription factor in hybrid poplars: A comparative study in trees growing under controlled and natural conditions
Source: PLoS One. 2017 Apr 4;12(4):e0174748. doi: 10.1371/journal.pone.0174748 (PMC5380328; doi:10.1371/journal.pone.0174748)
Supplement: S1 Table — (DOCX) [file pone.0174748.s001.docx]

**S1 Table. Primers used in this study.**

| Primer | Sequence |
| --- | --- |
| Dof5-F | 5´-GCATCAGAACGTCACTGG-3 |
| Dof5-R | 5´-CCTCTGCCATTCGAGACC-3 |
| PtPAL-F | 5´-CTGTCCTTACAGTTTCTTGGC-3 |
| PtPAL-R | 5´-CCACCTTGATCTCCCTTTCAA-3 |
| pcrFAD-F | 5´-GCAATGCCACCATTAACTGCCC-3 |
| pcrFAD-R | 5´-CAGCCTGCGCCATCAAATTTCC-3 |
| pcrDof5-F | 5´-TGGAGCAGAGGCTTAAGCATGC-3 |
| pcrDof5-R | 5´-TCCTCCTGTAAAGATGGCCACG-3 |
| PtFAD-F | 5´-ATGCTTCTTTCTGATACTTTAAC-3 |
| PtFAD-R | 5´-TCATCTATTTATGTTCTTCCAAG-3 |
| PpDof5-F | 5´-ATGAACTTAGCCTTAGCTCC-3 |
| PpDof5-R | 5´-TCATGGCAAACTGCTCCCTG-3 |
| PtGS1.3-F | 5´-TGGAAACCATAAGAGATCACCACC- 3´ |
| PtGS1.3-R | 5´- GAAGAGGCAATTCTTGTACCAAG- 3´ |
| PtDof19-F | 5´- GGGGAGGATTTGAATGCGGG-3 |
| PtDof19-R | 5´- GCAAGACCAGGCCACCCATT-3 |
| PtDof4-F | 5´- GGAGTCAGAGCCAATGGAATG-3 |
| PtDof4-R | 5´- CAATTTCCATAGTCTCTCAGAC -3 |
| UBQ-F | 5´- GTTGATTTTTGCTGGGAAGC- 3´ |
| UBQ_R | 5´- GATCTTGGCCTTCACGTTGT- 3´ |
| ACT-F | 5´- CCCATTGAGCACGGTATTGT-3´ |
| ACT-R | 5´- TACGACCACTGGCATACAGG- 3´ |
