# Supplementary figures and images for: Overexpression of a pine Dof transcription factor in hybrid poplars: A comparative study in trees growing under controlled and natural conditions
Source: PLoS One. 2017 Apr 4;12(4):e0174748. doi: 10.1371/journal.pone.0174748 (PMC5380328; doi:10.1371/journal.pone.0174748)

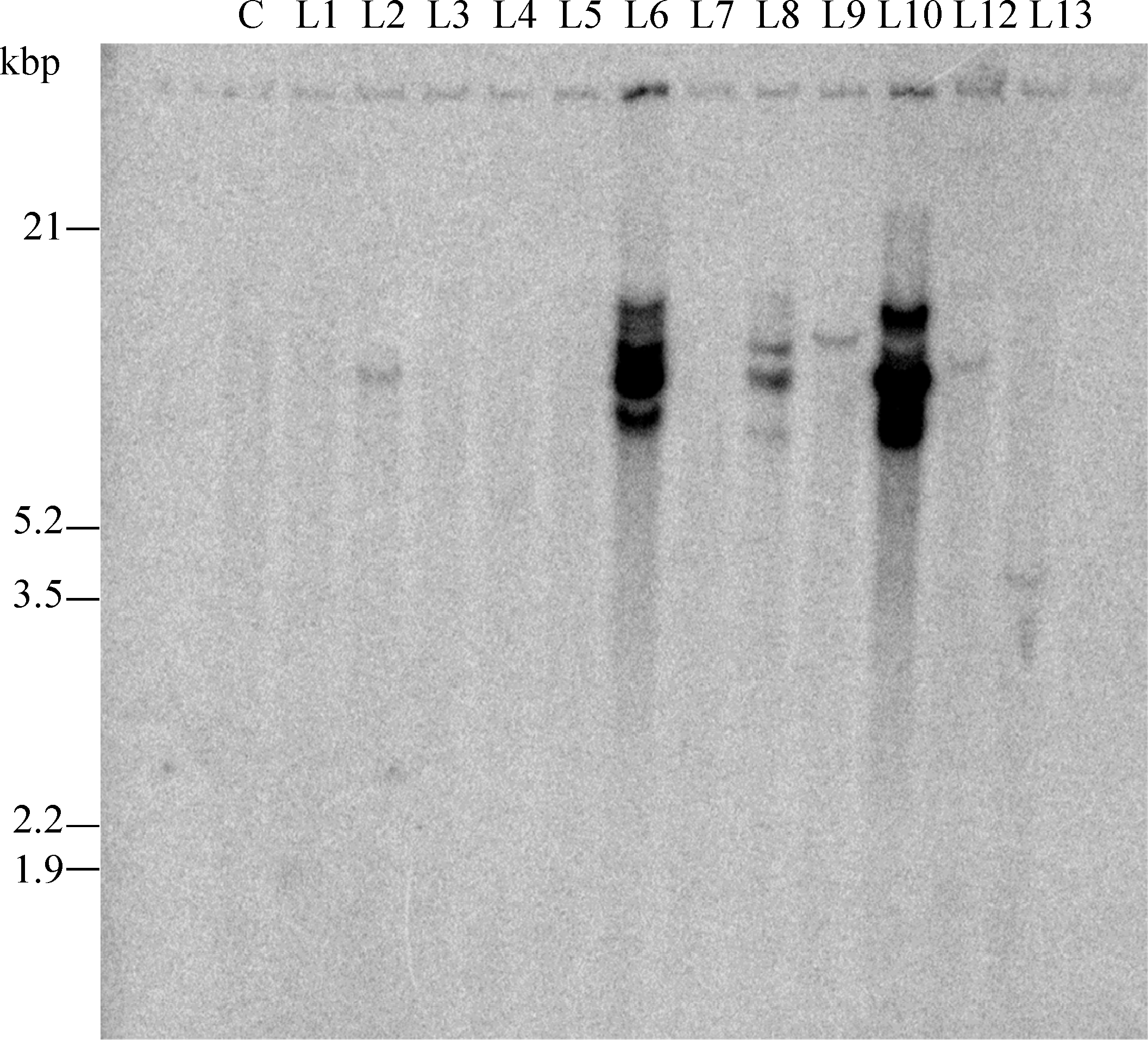

Supplement: S1 Fig — 10 μg of genomic DNA from control plant (C) and each line (L1-L10, L12 and L13) were digested with BamHI and separated on an agarose gel. Blots were hybridized with 32-P labelled PpDof5 cDNA. (TIF) [file pone.0174748.s002.tif]

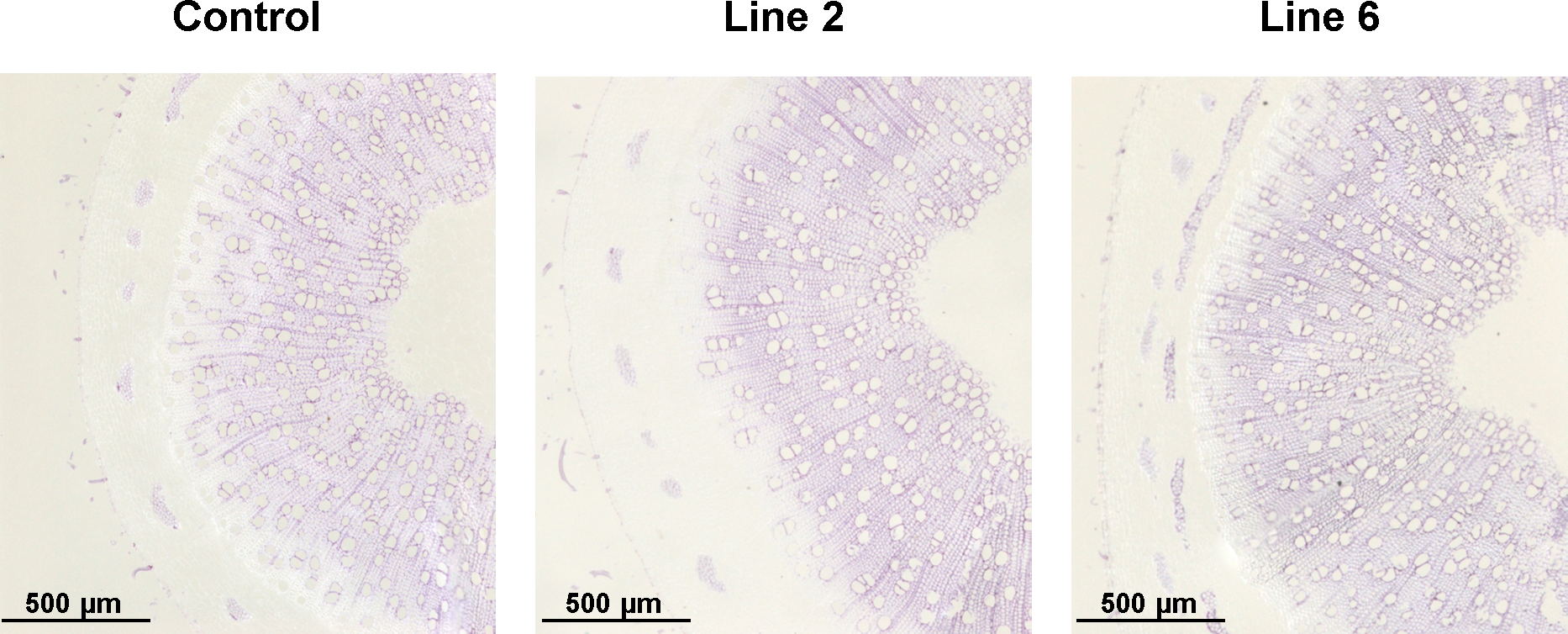

Supplement: S2 Fig — Cross-sections (10 μm thick) of control and transgenic lines were processed as described in Materials and Methods. The sections of stem were stained with phloroglucinol-HCl solution for detecting lignin. (TIF) [file pone.0174748.s003.tif]

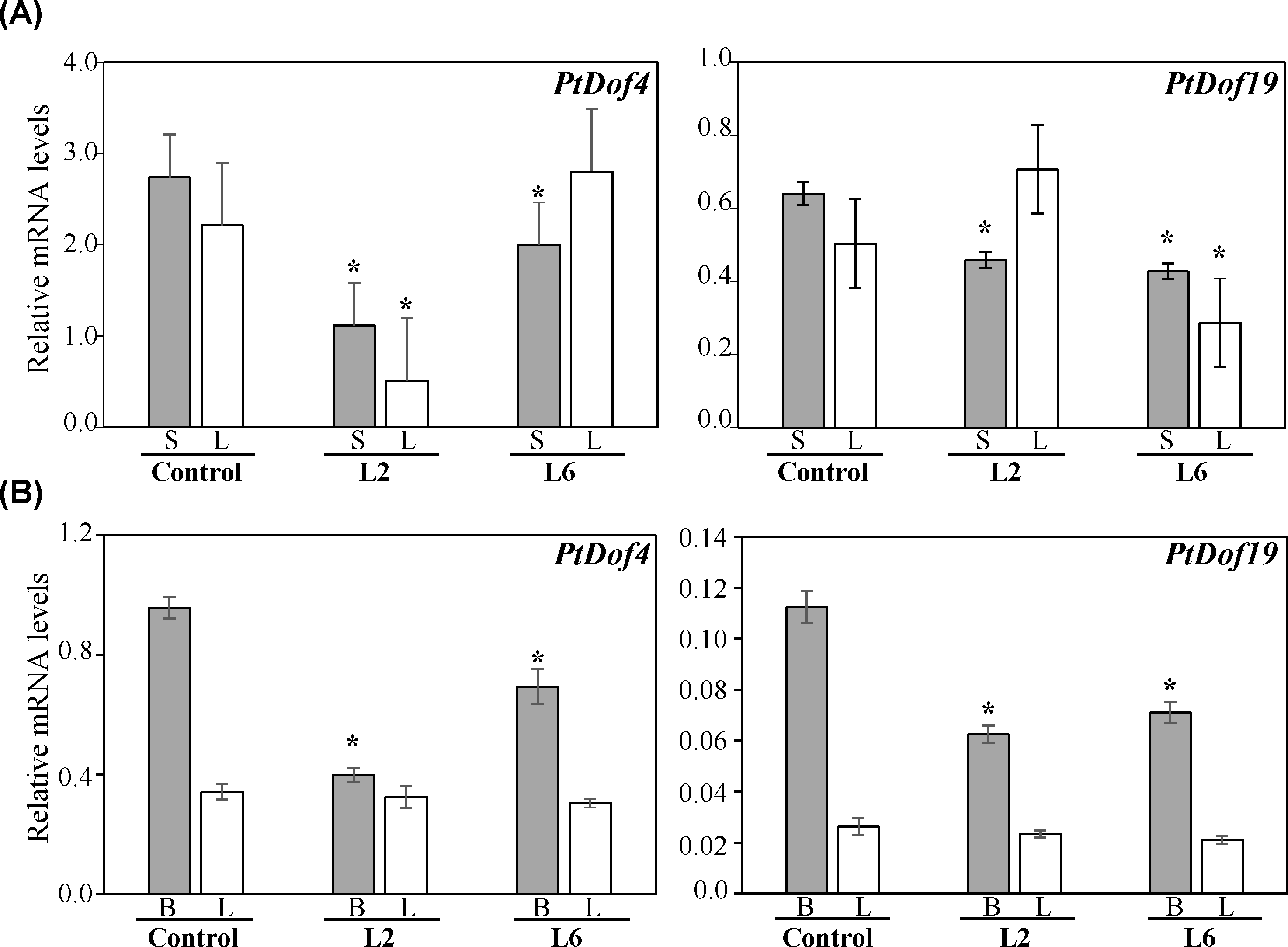

Supplement: S4 Fig — (A) Transcript levels were determined in stem (S) and leaves (L) of 10-weeks-old control hybrid poplar and L2 and L6 transgenics plants grown in a growth-chamber by qPCR using specific primers (S1 Table). (B) Transcript levels in side branches (B) and leaves (L) of poplar trees grown in the field. The expression data were normalized using Ubiquitin and actin as reference genes. Data represent the mean ± standard error of three technical replicates. Asterisks indicate that the difference between the control and transgenic plants was significant by the t-test (P< 0.05). (TIF) [file pone.0174748.s005.tif]
